# Explainable AI for Oral Cancer Diagnosis: Multiclass Classification of Histopathology Images and Grad-CAM Visualization

**DOI:** 10.3390/biology14080909

**Published:** 2025-07-22

**Authors:** Jelena Štifanić, Daniel Štifanić, Nikola Anđelić, Zlatan Car

**Affiliations:** 1 Faculty of Engineering, University of Rijeka, Vukovarska 58, 51000 Rijeka, Croatia; jelena.stifanic@uniri.hr (J.Š.); nikola.andjelic@uniri.hr (N.A.); 2 Faculty of Engineering, Catholic University of Croatia, Ilica 244, 10000 Zagreb, Croatia; zlatan.car@unicath.hr

**Keywords:** artificial intelligence, explainable deep learning, GRAD-Cam, histopathology images, oral squamous cell carcinoma

## Abstract

Oral cancer is typically diagnosed through histological examination; however, the primary issue with this type of procedure is tumor heterogeneity, where a subjective aspect of the examination may have a direct effect on the treatment plan for a patient. To reduce inter- and intra-observer variability, artificial intelligence algorithms are often used as computational aids in tumor classification and diagnosis. This research proposes a two-step approach for automatic multiclass grading using oral histopathology images (the first step) and Grad-CAM visualization (the second step) to assist clinicians in diagnosing oral squamous cell carcinoma. The Xception architecture achieved the highest classification values of 0.929 (±σ = 0.087) AUC_macro_ and 0.942 (±σ = 0.074) AUC_micro_. Additionally, Grad-CAM provided visual explanations of the model’s predictions by highlighting the precise areas of histopathology images that influenced the model’s decision. These results emphasize the potential of integrated AI algorithms in medical diagnostics, offering a more precise, dependable, and effective method for disease analysis.

## Simple Summary

An AI-based approach for diagnosing oral squamous cell carcinoma is developed using a database of histopathology images obtained through biopsy and evaluated by two pathologists. To increase the objectivity and repeatability of the histopathological examination, automated multiclass grading of OSCC is performed in the first step. Furthermore, the second step builds confidence in the AI-based system by integrating explainable AI components like Grad-CAM, which give clinicians valuable visual insights into the model’s decision-making process. Regarding multiclass grading, the method based on deep convolutional neural networks produced satisfactory results.

## 1. Introduction

Worldwide, the incidence of oral cancer (OC) has an increasing rate, which makes up 1–2% of all cancers [1,2]. OC has a high mortality rate, mainly due to late-stage diagnosis when tumor metastasis occurred. The five-year survival rate for early-stage cancer is approximately 80%, compared to the five-year survival rate at advanced stages, which is about 20% [3]. Over 90% of OC comprises oral squamous cell carcinoma (OSCC) [4].

Despite advances in medicine over the past decades, there are no clinical markers or other diagnostic tools that are useful in detecting cancerous change at the early stage [5]. Currently, standard methods for detecting oral cancer and the gold standard are clinical examination, conventional oral examination (COE), and histopathological evaluation following biopsy, which can detect cancer in the stage of established lesions with significant malignant changes [6,7]. However, the subjective nature of the examination is the primary issue when employing histological examination for tumor distinction, respectively, inter- and intra-observer variability [8]. Moreover, many other factors influence manual evaluation. These could include the condition of the microscope, lighting during the observation, the quality of the stains or slides, the amount of time spent on each observation, and so forth [9]. For that reason, reducing inter- and intra-observer variability and increasing objectivity and reproducibility using deep learning (DL) models could directly affect patient-specific therapeutic interventions by utilizing clinic-pathological features to determine patient outcomes [10].

For the detection and classification of OC, deep learning models have become an extremely powerful tool [11,12,13]. The ability of DL models to automatically extract features from images makes them suitable for medical image analysis. However, many DL models used for OC diagnosis are thought of as “black boxes”, which makes it challenging to comprehend their mechanisms to make decisions, especially in crucial applications like cancer diagnosis [14]. Therefore, creating DL models for OC analysis that are more reliable, interpretable, and clinically useful is crucial. Furthermore, there is a need for an OC classification model that provides explainable insights to improve trust and transparency in the diagnostic process and achieves high accuracy and sensitivity to ensure prompt detection of OC [15].

This research proposes OC multiclass classification using gradient-weighted class activation mapping (Grad-CAM) along with the DL model. By emphasizing the parts of the histopathological images that most influence the model’s predictions, this method offers explicable insights and improves the diagnostic process’s transparency and trustworthiness.

The main contributions of this research are as follows:

- a DL model for oral squamous cell carcinoma multiclass grading, which may enhance the objectiveness and repeatability of histopathological analysis and reduce the amount of time required for pathological inspections,
- improving trust and transparency in the AI-based diagnostic process by providing comprehensible insights utilizing Grad-CAM.

**Figure 1:**
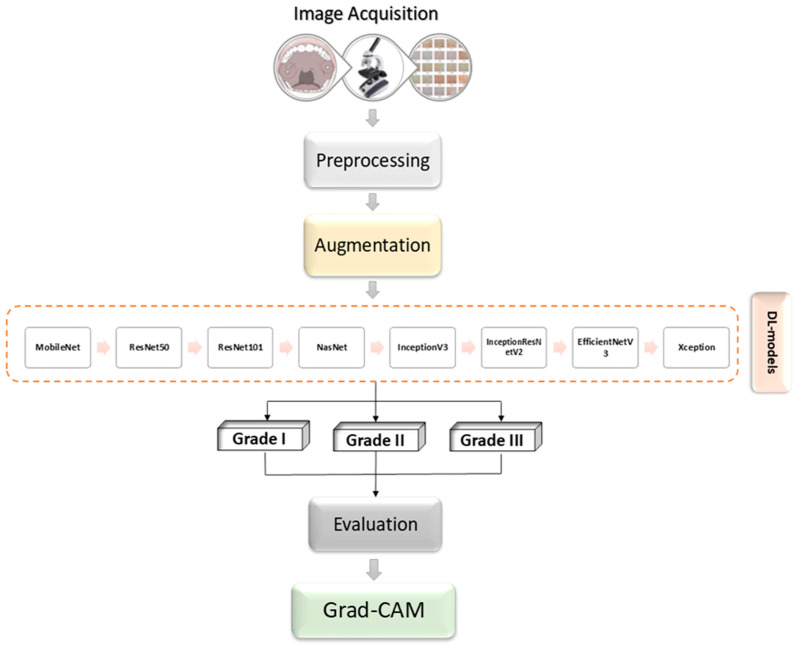
Block diagram illustration of the proposed methodology.

The methodology employed in this research focuses on an extensive analysis of oral cancer images. The procedure involves several steps, including preprocessing and augmenting the data, integrating multiple pretrained AI networks, and utilizing visualization tools like Grad-CAM. Figure 1 provides an overview of the proposed framework.

### Related Work

This section summarizes the current Artificial Intelligence (AI) solutions in a related field. It offers a brief review of the numerous models, approaches, and techniques applied in diverse solutions in related research fields.

According to the literature, most researchers have used DL models in retrospective studies to classify oral cancer. OC classification, often referred to as binary classification, establishes whether the given data is cancerous. Furthermore, the different stages (grades) of cancer can also be identified using multiclass classification.

Rahman et al. (2022) used a modified convolutional neural network (CNN) AlexNet to predict malignant and normal oral tissue based on biopsy images of oral squamous cell carcinoma [16]. As a result, the prediction accuracy and loss rate of the proposed model were 90.06% and 9.08%, respectively [16]. A system called OralNet is proposed by Mohan et al. (2023) for the detection of oral cancer from histopathology images [17]. The research is divided into four steps: The first step is to collect and prepare histopathology images. The second step involves extracting relevant features from images using both traditional and deep learning techniques. The third step includes feature reduction using the artificial hummingbird algorithm (AHA) and concatenation. The last step includes three-fold cross-validation performance validation and binary classification. According to test results, OralNet could identify oral cancer with above 99.5% accuracy [17]. Based on transfer learning and deep ensemble learning, Das et al. (2024) presented a classification model for binary oral cancer classification using histopathology images [18]. Ensemble learning can enhance the benefits of the DL technique by increasing generalization and accuracy. Using the stacking method, an ensemble model is built in this study that achieves 97.88% accuracy, exceeding base models [18].

Explainable AI (XAI) has drawn much attention, especially in fields like medical imaging, where precise and comprehensible machine learning models are essential for efficient diagnosis and treatment planning [19]. To improve interpretability and confidence in the outcomes, Grad-CAM is a baseline that identifies the most important image areas that are employed in a deep learning model’s decision-making process. It is used for various computer vision (CV) applications, including explanation and classification [19].

Several studies have employed Grad-CAM to classify cancer images using higher accuracy, precision, and recall in order to increase diagnostic reliability and interpretability.

Oya et al. (2023) aimed to examine AI’s capacity to assess OSCC using a novel training approach that considers cellular and structural atypia and their applicability [20]. The model of the convolutional neural network used was EfficientNetB0. Its validity was clarified using gradient-weighted class activation mapping. The suggested technique used images with 512 × 512 pixels as input and reached a high accuracy of 99.65%. According to Grad-CAM results, AI model concentrated on the area around the basal layer and addressed both cellular and structural atypia of OSCC [20]. In order to predict oral squamous cell carcinoma, Afify et al. (2023) paper suggests a novel model that uses deep transfer learning and gradient-class activation mapping to identify the lesion area in the histopathology image [21]. The results obtained from the proposed approach are significant because they represent the clinical community’s critical leadership in the early, accurate detection of oral cancer [21]. In order to gain an improved comprehension of the capabilities and limitations of DL methods in the context of oral cancer diagnosis, Da Silva et al. (2024) conducted a comprehensive evaluation of the performance of two DL models that are well-known for their high accuracy in oral cancer classification. Their analysis went beyond simple accuracy metrics; they used Grad-CAM to provide visual explanations of the models’ decisions, and they also looked into subclass accuracy rates and the distribution of prediction confidences [22].

A thorough review of the literature shows that, at the time when this research was performed, no work has been done on multiclass classification along Grad-CAM visualization using histopathology images obtained by biopsy and stained with marker protein.

## 2. Materials and Methods

This section provides a detailed description of the dataset used for OSCC classification as well as a brief overview of Grad-CAM technique. Furthermore, a description of deep learning models and evaluation criteria are described.

### 2.1. Dataset Description

**Figure 2:**
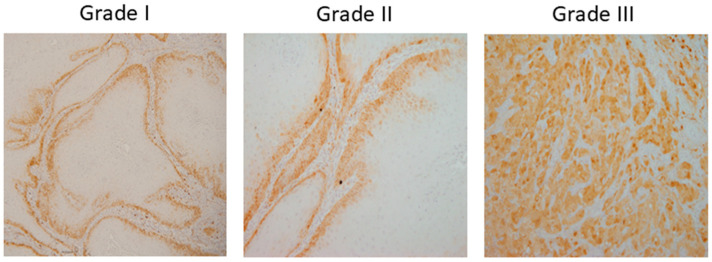
OSCC group of Grade I (well-differentiated), Grade II (moderately differentiated) and Grade III (poorly differentiated).

A dataset of 322 histology images with 768 × 768-pixel size was created for this research. The formalin-fixed, paraffin-embedded oral mucosa tissue blocks of histopathological reported cases of OSCC were retrieved from the archives of the Clinical Department of Pathology and Cytology, Clinical Hospital Center in Rijeka. Sample slides were examined and classified in accordance with World Health Organization (WHO) guidelines by two unbiased pathologists [23]. The level of agreement among the pathologists was assessed using the Kappa coefficient. The Kappa coefficient was determined to be 0.94. Images were divided into three classes: Grade I (well-differentiated), Grade II (moderately differentiated) and Grade III (poorly differentiated), as shown in Figure 2.

Briefly, 4 µm sized paraffin-embedded tissue sections were stained following standard immunohistochemistry (IHC) protocol with various marker proteins. The IHC images used were stained with DAB and hematoxylin. Images were captured using the light microscope (Olympus BX51, Olympus, Tokyo, Japan) equipped with a digital camera (DP50, Olympus, Japan) and transmitted to a computer by CellF software (Olympus, Japan, https://www.olympus.co.jp/). Furthermore, images were captured with 10× objective lenses.

A comparable clinic-pathological report of the patients is shown in Table 1. The patient’s age at the time of diagnosis, sex, smoking status, and alcohol consumption were among the demographic details. The patients were adults with a median age of 64. While 38% of the patients consumed alcohol, 55% of them smoked. 30% of the patients were female, and 70% were male. Only 15% of patients had a grade III diagnosis, whereas 45% received a grade I diagnosis. A higher percentage of patients (52%) had lymph node metastases.

To achieve good performance and prevent overfitting, Deep Convolutional Neural Networks require a large number of samples. Since some fields, like medical image analysis, do not always have access to many samples the data augmentation is necessary.

By performing data augmentation techniques, the number of samples can be artificially increased. The following geometrical transformations are employed in the augmentation process: 90-, 180-, and 270-degree anticlockwise rotations; vertical flip; vertical flip combined with 90-degree anticlockwise rotation; horizontal flip; and horizontal flip combined with 90-degree anticlockwise rotation. The augmentation process only serves to create training samples since newly generated data are variants of the original data, testing samples are not augmented.

**Figure 3:**
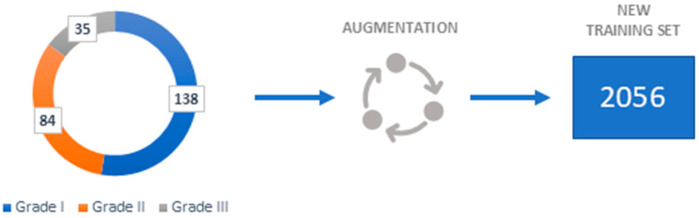
Dataset representation.

As seen in Figure 3, a new training set of 2056 images has been created using the aforementioned transformations.

### 2.2. Gradient Weighted Class Activation Mapping (Grad-CAM)

Unprecedented advances in a range of computer vision tasks, including semantic segmentation, object detection, and image classification, have been achieved through the use of deep neural models based on convolutional neural networks. Although these models allow for better performance, they are difficult to understand since they cannot be separated into separately intuitive components.

In order to make decisions from a broad class of CNN-based models more transparent and understandable, Selvaraju et al. (2017) proposed a method for creating “visual explanations” [24]. Their method, called Gradient-weighted Class Activation Mapping (Grad-CAM), produces a coarse localization map that highlights the key areas in the image for concept prediction by using the gradients of any target concept flowing into the final convolutional layer.

To construct the class discriminative localization map $L_{Grad-CAM}^{c}\in R^{uxv}$, the authors first calculate the gradient of the class score c, $y^{c}$ with respect to feature maps $A^{k}$. Global average pooling gradients are used to determine the neuron significance weights, $\alpha_{k}^{c}$:

(1) $$\alpha_{k}^{c}=\frac{1}{Z}\sum_{i}\sum_{j}\frac{\partial y^{c}}{\partial A_{ij}^{k}}$$

This represents the significance of feature map *k* for a target class *c* and represents a partial linearization of the deep learning model downstream from *A*. By using the ReLU activation function, $\alpha_{k}^{c}$ gathers the corresponding class discriminative localization map;

(2) $$L_{Grad-CAM}^{c}=ReLU\left(\sum_{k}\alpha_{k}^{c}A^{k}\right)$$

In general, a CNN that classifies images does not always need to produce $y^{c}$ as its class score. It might be any differentiable activation, such as a question response or words from a caption.

The global average pooling is used to spatially pool the K feature maps $A^{k}\in R^{uxv}$. The pooled feature map and linear transformation are then used to obtain the class *c* score, $S^{c}$:

(3) $$S^{c}=\sum_{k}w_{k}^{c}\frac{1}{Z}\sum_{i}\sum_{j}A_{ij}^{k}$$

It is possible to modify the Equation (3) by using $L^{c}$ (CAM):

(4) $$S^{c}=\frac{1}{Z}\sum_{i}\sum_{j}\sum_{k}w^{c}A_{ij}^{k}$$

### 2.3. Deep Learning Models

Combining medical image analysis with deep learning models allows for the real-time analysis of large and complex medical datasets, providing insights that improve patient outcomes. Such algorithms can be employed to classify and distinguish between different stages of a disease. This subsection gives an overview of standard image classification algorithms:

- ResNet—Since training deep neural networks is challenging, He et al. (2016) introduce a residual learning system for training networks significantly deeper than previously used networks [25]. They evaluated residual nets with a depth of up to 152 layers on the ImageNet which resulted in a 3.57% error [25]
- MobileNetv2—Sandler et al. (2018) describe a new mobile architecture called MobileNetv2. Their basic building unit has many characteristics that make it especially well-suited for mobile applications [26]. The described architecture enhances the state-of-the-art for a wide range of performance points on the ImageNet dataset [26].
- Xception—Chollet (2017) demonstrated a new deep convolutional neural network architecture inspired by Inception called Xception [27]. Inception modules are replaced with depth-wise separable convolutions in this architecture, which results in higher performance improvement [27].
- EfficientNet—In their paper, Tan and Lee (2019) propose a novel scaling method called EfficientNet [28]. To scale up CNNs in a more structured manner, such a method employs a simple yet highly effective compound coefficient. EfficientNets, by significantly improving model efficiency could potentially serve as a new foundation for future computer vision tasks, according to the authors [28].
- InceptionV3—The concept of InceptionV3 was put forth by Szegedy et al. (2016) after InceptionV1 and InceptionV2. Its main goal is to reduce processing power by altering earlier Inception architectures. Several network optimization methods, including factorized convolutions, regularization, dimension reduction, and parallelized calculations, have been proposed in InceptionV3 that loosens the constraints for more straightforward model adaptation [29].
- InceptionResNetV2—InceptionResNetv2, which combines the Inception design with residual connections, was developed by Szegedy et al. (2017) since it has been proven that the Inception architecture produces good results at a comparatively cheap computational cost. The presented architecture significantly increased training speed and enhanced recognition performance [30].
- NASNet—In their research, Zoph et al. (2018) demonstrated a method for directly learning model architectures on the relevant dataset. Since this approach is costly when the dataset is large, they propose utilizing a small dataset to identify an architectural building block that can subsequently be applied to a larger dataset. Designing a new search space that allows for transferability, which researchers refer to as the “NASNet search space” is the main contribution of their work [31].

### 2.4. Evaluation Criteria

Statistical metrics like the micro- and macro-Area Under the Curve (AUC) are used to assess the classification performance of models since the aim of this research is to perform multiclass classification of OSCC grades. Micro and macro averaging can be used to determine AUC_micro_ and _-macro_, respectively. A micro averaging of the true positive rate (TPR) uses the total number of samples as the denominator and the number of valid classifications for each class as the numerator. Moreover, the fallout or false positive rate (FPR) is computed as the ratio of false classifications for each class to the total number of samples [32]. Micro averaging is represented mathematically as follows:

(5) $${TPR}_{micro}=\frac{\sum_{i=1}^{k}{TP}_{i}}{\sum_{i=1}^{k}{TP}_{i}+\sum_{i=1}^{k}{FN}_{i}}$$

and

(6) $${FPR}_{micro}=\frac{\sum_{i=1}^{k}{FP}_{i}}{\sum_{i=1}^{k}{FP}_{i}+\sum_{i=1}^{k}{TN}_{i}},$$

where:

- true positive (TP) is when both the predicted and actual values are positive,
- true negative (TN) is when both the actual and predicted values are negative,
- false negative (FN) is when a negative prediction is made but the actual number is positive and,
- false positive (FP) is when a prediction is positive, but the actual value is negative [33].

After calculating the metrics for every class independently, macro averaging for k classes averages the outcomes. To compute AUC_macro_, a TPR_macro_ and FPR_macro_ are utilized, which can be expressed as:

(7) $${TPR}_{macro}=\frac{\sum_{i=1}^{k}{TPR}_{i}}{k}$$

and

(8) $${FPR}_{macro}=\frac{\sum_{i=1}^{k}{FPR}_{i}}{k}.$$

The AUC value is defined between 0 and 1.0, where a higher value indicates better performance of the model and vice-versa. Additionally, stratified *k*-fold cross-validation is utilized as a statistical method in order to evaluate the performance of the models used in this research. Such an approach ensures more stable estimate and generalization since the evaluation is performed multiple “*k*” times on different portions of the dataset. The main difference between the classical *k*-fold and stratified *k*-fold cross validation is that stratified *k*-fold preserves original class distribution within each fold. Aforementioned is crucial especially in imbalanced datasets [34]. In order to prevent data leakage, the authors implemented strict patient-level separation during cross-validation. Such an approach ensures that all samples from a single patient are contained within the same fold, therefore preventing any patient’s data from appearing in both training and validation sets simultaneously.

## 3. Results and Discussion

In this section, the experimental results of the proposed methodology are shown. Using the pretrained ResNet50, ResNet101, NASNet, Xception, InceptionV3, MobileNetV2, InceptionResNetV2 and EfficientNetB3 architectures on ImageNet, initial experimental results are obtained. At the top of each aforementioned architecture, two additional layers are added in order to enable multiclass classification of the OSCC grades. The first layer is the global average pooling layer, which reduces the h×w×c (height, width, channels) tensor to a 1×1×c, which also forces the network to focus on global spatial information. Furthermore, the fully connected layer is the second added layer, consisting of three neurons and a Softmax activation function, as the focus of this research is to perform OSCC grading in three classes: Grade I, Grade II, and Grade III.

Stochastic Gradient Descent (SGD), Adam, and RMSprop are the three optimizers used to train each model architecture. Additionally, each model architecture is trained in two phases; in the first phase only the output layer is trainable while the rest was frozen, and the second phase in which the output layer is frozen while all the other layers are trainable. Such an approach ensures gradual adaptation and stable training.

**Figure 4:**
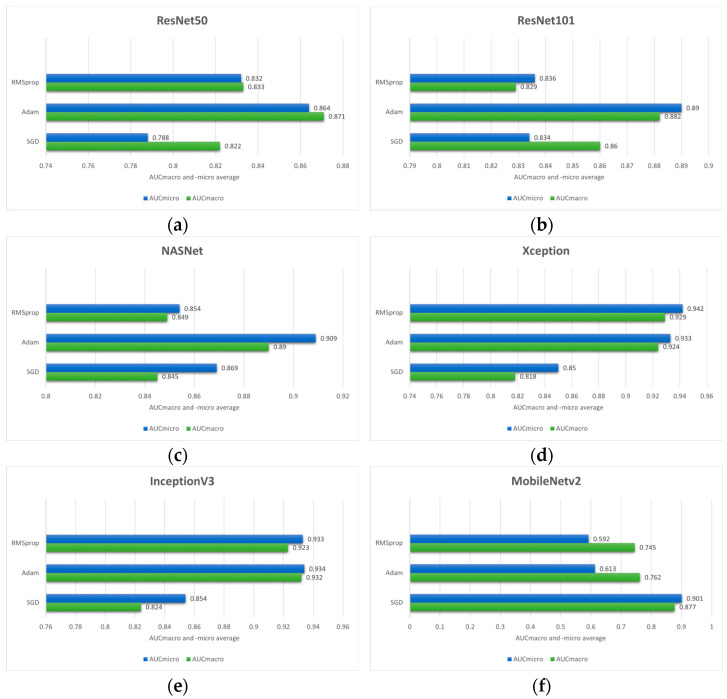

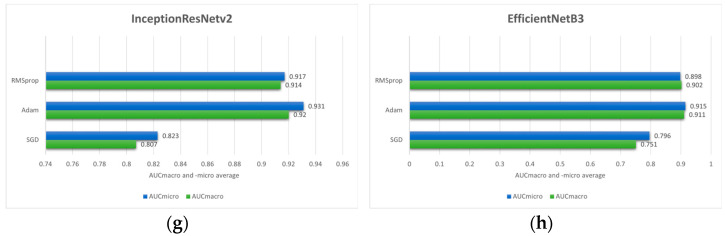
Comparison of the mean AUC_macro_ and _-micro_ values of three different optimizers (RMSprop, Adam and SGD) on pre-trained models: (**a**) ResNet50; (**b**) ResNet101; (**c**) NASNet; (**d**) Xception; (**e**) InceptionV3; (**f**) MobileNetV2; (**g**) InceptionResNetV2 and (**h**) EfficientNetB3.

By utilizing early stopping and adjusting optimizer hyperparameters such as learning rate and learning rate decay, the results shown in Figure 4. are obtained. Additionally, stratified 5-fold cross-validation is employed since it provides a robust and unbiased estimate of model performance.

Analyzing the experimental results, the overall highest values of performance measure are achieved when, in the first phase, only the output layer is trained with a learning rate of 1 × 10^−3^ and a learning rate decay of 1 × 10^−6^. Moreover, in the second phase, the output layer was frozen, and the remaining model layers were trained with learning rate and learning rate decay values of 1 × 10^−4^ and 1 × 10^−6^, respectively.

Based on the outcome of the stratified 5-fold cross-validation it can be seen that Adam optimizer provides the superior performance across most of the model architectures according to AUC_macro_ and _-micro_ values. Therefore, in the case of this research, the Adam optimizer can be considered as a more effective optimization strategy compared to SGD and RMSprop. In the case of the ResNet50 model architecture, the highest values of performance measures are achieved utilizing the Adam optimizer with the values of 0.871 ± 0.105 (AUC_macro_) and 0.864 ± 0.090 (AUC_micro_). On the other hand, the SGD yielded the lowest performance values. Similarly, ResNet101 model architecture trained using Adam resulted in AUC_macro_ and AUC_micro_ values of 0.882 ± 0.125 and 0.890 ± 0.112, respectively. Moreover, the NASNet model architecture combined with Adam optimizer yielded the highest performances (0.890 ± 0.054 AUC_macro_ and 0.909 ± 0.043 AUC_micro_). On the contrary, RMSprop provided the lowest values of performance measures implying that optimizer learning strategy is less compatible with the data used in this research along with the feature representations learned by the model itself.

Interestingly, RMSprop combined with Xception architecture yielded the highest overall performance with the values of 0.929 ± 0.087 and 0.942 ± 0.074 for the AUC_macro_ and AUC_micro_, respectively. This strongly implies that the effectiveness of the optimizer can be model architecture dependent. In the case of IncetionV3, the highest AUC_macro_ and AUC_micro_ are achieved with the values of 0.932 ± 0.081 and 0.938 ± 0.088, while the SGD produced the lowest values. Moreover, when combined with SGD optimizer, MobileNetV2 model architecture achieved the highest AUC_macro_ value of 0.877 ± 0.062 and AUC_micro_ value of 0.901 ± 0.049. On the other hand, the highest AUC_macro_ of 0.920 ± 0.059 and AUC_micro_ of 0.931 ± 0.0.064 are obtained by utilizing the Adam optimizer with InceptionResNetV2 model architecture, while the lowest performance measure resulted when InceptionResNetV2 was combined with SGD optimizer. EfficientNetB3 architecture, the last one shown in Figure 3, indicates the highest AUC_macro_ of 0.911 ± 0.148 and AUC_micro_ of 0.915 ± 0.148 when is combined with Adam optimizer. In addition to high performance values, the standard deviation is also relatively larger compared to other model architectures, indicating greater variability across the 5-folds.

According to the presented performances of the model architectures and optimizers, it can be seen that in most cases the Adam optimizer provides high classification performance on data used in this research. While RMSprop provided mixed results, excelling only with Xception model architecture, SGD underperforms in most cases.

In order to establish performance benchmarks, two additional conventional machine learning algorithms are employed as baseline models to perform the classification of histopathology images into three classes (Grade I, Grade II, and Grade III). The logistic regression classifier, implemented with a limited-memory Broyden-Fletcher-Goldfarb-Shanno (L-BFGS) solver, resulted in AUC_macro_ of 0.509 ± 0.060 and AUC_micro_ 0.634 ± 0.059. On the other hand, the k-nearest neighbors (KNN) configured with 5 nearest neighbors yielded in AUC_macro_ of 0.539 ± 0.052 and AUC_micro_ of 0.658 ± 0.035. A comprehensive comparison of the baseline models with the deep learning model architectures is presented in Table 2.

In order to enable an unbiased evaluation of the efficacy of utilized models tested under the same experimental settings, a statistical analysis (Friedman test) is performed to ascertain whether there are statistically significant differences in their performances. The analysis is conducted under the null-hypothesis that no significant difference exists in model performance, implying equivalent performance across all evaluated model architectures. The test yielded a Friedman statistic of 28.354 with a corresponding *p*-value of 0.0008. The *p*-value in this case is significantly below the threshold of 0.05 indicating a statistically significant difference in performance among evaluated models, thereby allowing rejection of the null-hypothesis that all models perform equivalently.

Furthermore, to identify specific pairwise differences between models, post-hoc analysis is conducted utilizing the Nemenyi test. The results revealed several statistically significant pairwise comparisons at the 5% significance level. Specifically, logistic regression performed significantly differently from Xception (*p*-value of 0.0169) and InceptionV3 (*p*-value of 0.0338). Additionally, KNN also differed significantly from Xception with the *p*-value of 0.0338. The acquired test results suggest that deep learning model architectures achieved consistently superior performance compared to traditional baseline classification methods.

The remaining pairwise comparisons did not yield statistically significant differences, indicating comparable performance among the remaining models at the 5% significance level. Notably, the lack of statistical significance does not rule out the existence of practically meaningful differences. Even if statistical significance is not achieved, consistent advantages in performance metrics, such as AUC, may nevertheless represent practically meaningful improvements.

**Figure 5:**
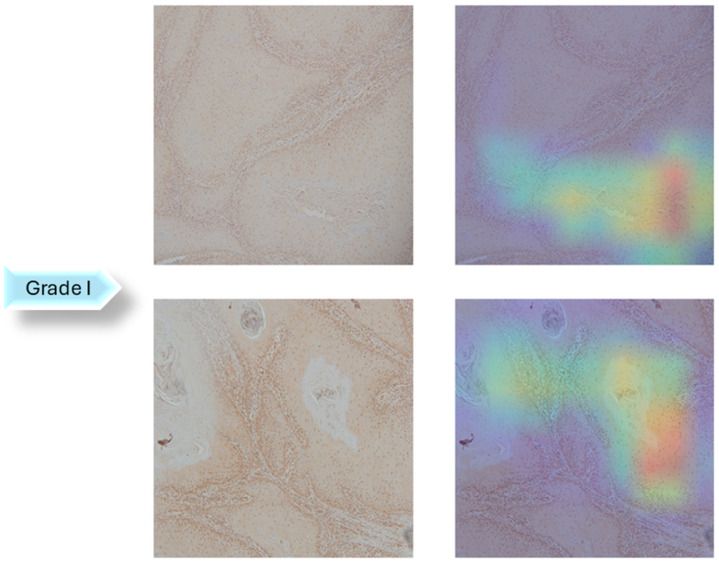
Grad-CAM Visualization of Grade I.

**Figure 6:**
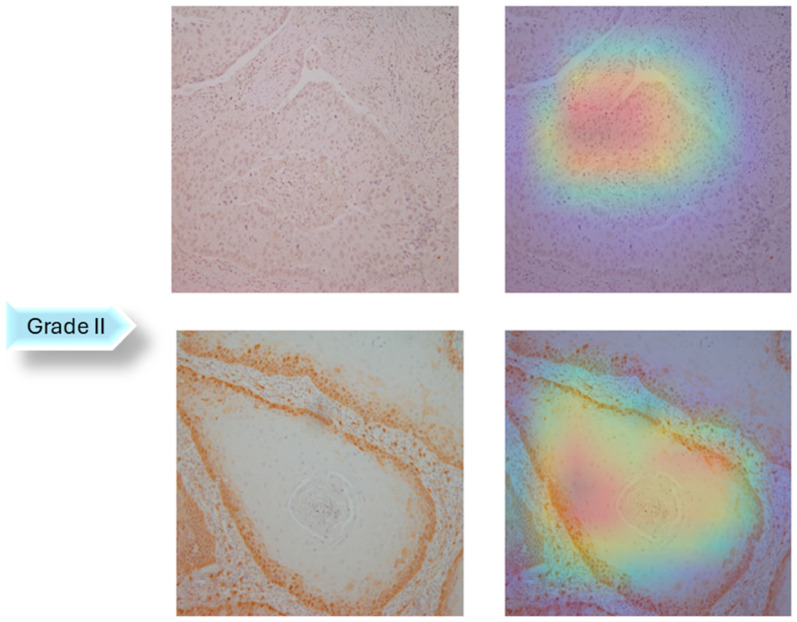
Grad-CAM Visualization of Grade II.

**Figure 7:**
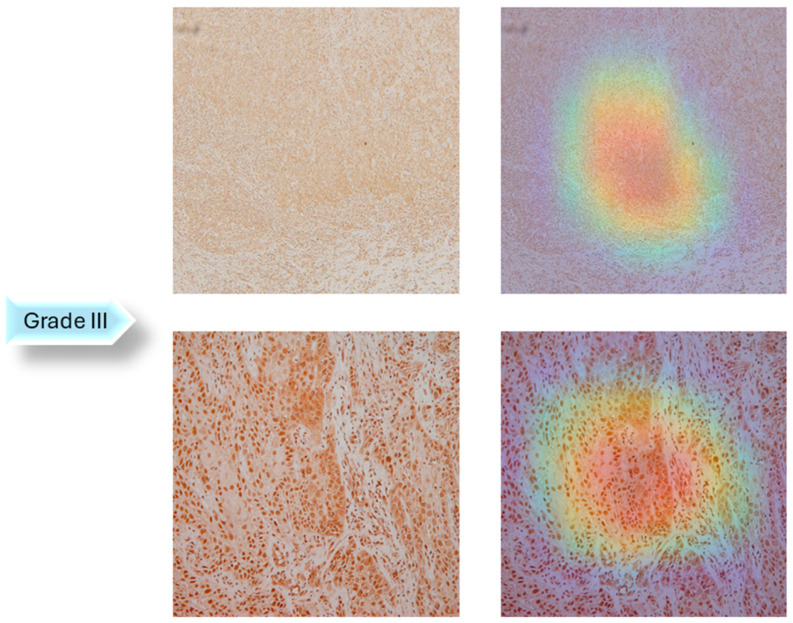
Grad-CAM Visualization of Grade III.

In the next step of the research, Gradient-weighted Class Activation Mapping was used to visually explain which regions of the images affected the model’s predictions. These visualizations provide another level of interpretability to the model’s decision-making process by enabling healthcare professionals to identify which histopathology slide findings are most suggestive of classifications. This can help with further diagnostic reasoning and increase confidence in automated systems. The Grad-CAM visualization of the proposed model is shown in Figure 5, Figure 6 and Figure 7. The color spectrum, which ranges from blue to red, clearly identifies the sections of an image that are most important for the model’s prediction output. The highest and lowest activation areas are represented by red and blue, respectively.

As seen in Figure 5, Figure 6 and Figure 7, Grad-CAM is utilized to create heatmaps that identify key areas in histopathology images for multiclass classification. It captures gradients related to specific output classes, such as Grade I, Grade II, and Grade III, that flow into the final convolutional layers. To create a localization map, these gradients are pooled channel-wise, emphasizing important regions for class prediction. Heatmaps are created by forward passing an image through the network, computing gradients in relation to feature maps, spatially pooling these gradients, and combining weights with activation maps. Clinically, these heatmaps help differentiate pathologically significant features from possible artefacts or irrelevant regions by visually validating the model’s focus on important regions. Grad-CAM helps health professionals make accurate decisions by improving the validation and transparency of AI-driven diagnostic analyses.

However, such an approach has several limitations that reduce its usefulness for understanding deep learning models. Grad-CAM primarily highlights high-level features from the convolutional neural network’s deeper layers. These layers often overlook crucial low- or mid-level features such as edges, textures, or fine-grained structures in favor of abstract patterns or class-discriminative areas. This is a drawback in medical imaging since minor features, such as microscopic lesions, microcalcifications, or early-stage abnormalities, often appear in earlier layers of the network and can be crucial for diagnosis. The spatial resolution is another drawback. The generated heatmaps are coarse since Grad-CAM employs feature maps from deeper layers, which are downsampled as a result of pooling procedures. For that, it is challenging to locate small but crucial parts in an image precisely.

Nevertheless, the upside of this technique is that the Grad-CAM can be used for purposes other than diagnosing oral cancer from histopathology images. It can be used with various medical imaging modalities, including ultrasounds, CT scans, MRIs, and X-rays, to produce heatmaps that emphasize important areas that affect model predictions. Furthermore, when it comes to tasks such as identifying lung, breast, brain tumors, or cardiovascular abnormalities, this interpretability can increase confidence in AI models [35,36,37].

With a standard deviation of ±σ = 0.087 and ±σ = 0.074, respectively, the Xception architecture with RMSprop as an optimizer achieved AUC_macro_ and AUC_micro_ more than 0.9. Such values of standard deviation and performance measure show that the model is highly robust, and when combined with Grad-CAM visualization, it has a lot of potential as an assistive tool for medical image analysis. Many disorders, such as Alzheimer’s, liver cirrhosis, or chronic lung diseases, require accurate localization of abnormalities within imaging data, much as oral cancer diagnosis. Healthcare personnel can benefit from the transparent nature of DL model predictions and Grad-CAM visual outputs by ensuring that the AI’s focus aligns with known pathology, which improves diagnostic accuracy in complex cases.

The proposed AI-based system can be used as a decision support tool integrated into the digital pathology pipeline. Following the scanning and preprocessing of digitized histopathology slides, the model may automatically identify regions of interest and provide classification results. Grad-CAM visualizations can also be used to highlight the image regions that had the most influence on the model’s decision. As visual aids, Grad-CAM heatmaps might be included in pathology reports to assist pathologists comprehend and assess the model’s findings. In situations that are ambiguous or borderline, heatmaps may be particularly helpful in highlighting particular histological features that demand further investigation.

AI is most suited as a supplemental tool to pathologists rather than as a replacement, especially in positions such as triage assistant or second reader, which improve diagnostic efficiency, accuracy, and consistency. An AI-based system can serve as a second reader and offer pathologists objective, real-time assistance by reducing cognitive and perceptual errors, particularly in complex or visually subtle cases. Furthermore, AI-based systems can be used as a triage mechanism to identify urgent or high-risk cases that require manual evaluation. In rare or unusual disease presentations, where AI can serve as a knowledge extender, it has shown the potential to reduce diagnostic time and error rate. Moreover, such systems have much potential to increase the effectiveness and precision of diagnostics, particularly in well-defined clinical subgroups.

## 4. Conclusions

This research highlights the enormous potential of applying XAI-based algorithms to improve OSCC diagnosis accuracy and survival rates. To attain satisfactory classification performance, the authors investigated multiple deep learning models utilizing different configuration settings for the multiclass classification task. According to the results, Xception architecture resulted in the highest classification performance, 0.929 AUC_macro_ and 0.942 AUC_micro_, with the lowest standard deviations, ±σ = 0.087 and ±σ = 0.074, respectively. In conclusion, this research show the importance of model architecture selection, optimizer selection and model-optimizer compatibility for the specific data and task in order to achieve optimal performance.

Additionally, Grad-CAM visualization was also used to validate interpretability to the model’s decision-making process. This approach outperforms traditional single-model approaches, offering a more thorough analysis with less variability and human error.

Since the research’s data availability was limited, a dataset with more histopathological images should be used in future research to produce a more reliable model. Furthermore, adding more oral cancer types to the dataset would enable the algorithm to identify additional morphological features and improve its applicability in a larger range of clinical settings. For a more thorough understanding of tumor biology, future research should also incorporate other modalities, such as molecular profiling and genomic data (such as TP53 mutations, genomic instability, and other driver alterations). These molecular processes may also be related to grading, differentiation, or response to treatment, and they frequently underline the morphological changes seen in tissue. Additionally, future work should incorporate risk factors into the AI model for a thorough and realistic model of OSCC progression. Such an approach can aid in understanding the ways in which lifestyle, histology, and demographic factors interact to affect the patient’s prognosis and the severity of the disease.

Prospective validation in actual clinical settings is a crucial next step to improve the beneficial relevance and generalizability of AI model. This would entail implementing the AI-based system in a diagnostic process, initially in a supportive capacity, enabling pathologists to compare their interpretations with AI-driven suggestions.

## Figures and Tables

**Table 1:** Characteristics of the patients include sex, age, smoking and alcohol habits, presence of metastases in the lymph nodes, and grade of oral squamous cell carcinoma.

Characteristic of the Patients		*n* = 40 (100%)
Sex	F	30
	M	70
Age	To 49	5
	50–59	13
	60–69	55
	+70	27
Smoking	Y	55
	N	45
Alcohol	Y	38
	N	62
Lymph Node Metastases	Y	52
	N	48
Grading	I	45
	II	40
	III	15

**Table 2:** Performance of different algorithms utilizing the stratified 5-fold cross-validation.

Algorithm	AUC_macro_ ± σ	AUC_micro_ ± σ
ResNet50	0.871 ± 0.105	0.864 ± 0.090
ResNet101	0.882 ± 0.125	0.890 ± 0.112
NASNet	0.890 ± 0.054	0.909 ± 0.043
Xception	0.929 ± 0.087	0.942 ± 0.074
InceptionV3	0.932 ± 0.081	0.938 ± 0.088
MobileNetv2	0.877 ± 0.062	0.900 ± 0.049
InceptionResNetV2	0.920 ± 0.059	0.931 ± 0.0.064
EfficientNetB3	0.911 ± 0.148	0.915 ± 0.148
Logistic Regression	0.509 ± 0.060	0.634 ± 0.059
KNN	0.539 ± 0.052	0.658 ± 0.035

## Data Availability

The data presented in this study are available on request from the corresponding author if data sharing is approved by the ethics committee. The data are not publicly available due to data protection laws and conditions stated by the ethics committee.

## Abbreviations

The following abbreviations are used in this manuscript:

OC	Oral cancer
OSCC	Oral squamous cell carcinoma
COE	Conventional oral examination
DL	Deep Learning
Grad-CAM	Gradient-weighted class activation mapping
AI	Artificial Intelligence
CNN	Convolutional neural network
AHA	Artificial hummingbird algorithm
XAI	Explainable Artificial Intelligence
CV	Computer vision
WHO	World Health Organization
IHC	Immunohistochemistry
AUC	Area Under the Curve
KNN	K-nearest neighbors
